# Why is preventing antibiotic resistance so hard? Analysis of failed resistance management

**DOI:** 10.1093/emph/eoaa020

**Published:** 2020-07-02

**Authors:** Shiwei Zhou, Camilo Barbosa, Robert J Woods

**Affiliations:** Division of Infectious Diseases, Department of Internal Medicine, University of Michigan, Ann Arbor, MI 48109-5680, USA

**Keywords:** translational evolutionary medicine, antibiotic resistance, clinical decision making, antibiotic stewardship

## Abstract

We describe the case of a patient with pancreatitis followed by intra-abdominal infection in which source control was not achieved. Antimicrobial therapy led to the emergence of resistance in multiple organisms through multiple population dynamics processes. While the initial insult was not due to infection, subsequent infections with resistant organisms contributed to a poor outcome for the patient. Though resistance evolution was a known risk, it was difficult to predict the next organism that would arise in the setting of antibiotic pressure and its resistance profile. This case illustrates the clinical challenge of antibiotic resistance that current approaches cannot readily prevent.

LAY SUMMARY

Why is antibiotic resistance management so complex? Distinct evolutionary processes unfold when antibiotic treatment is initiated that lead, separately and together, to the undesired outcome of antibiotic resistance. This clinical case exemplifies some of those processes and highlights the dire need for evolutionary risk assessments to be incorporated into clinical decision making.

## INTRODUCTION 

**Figure 1.**
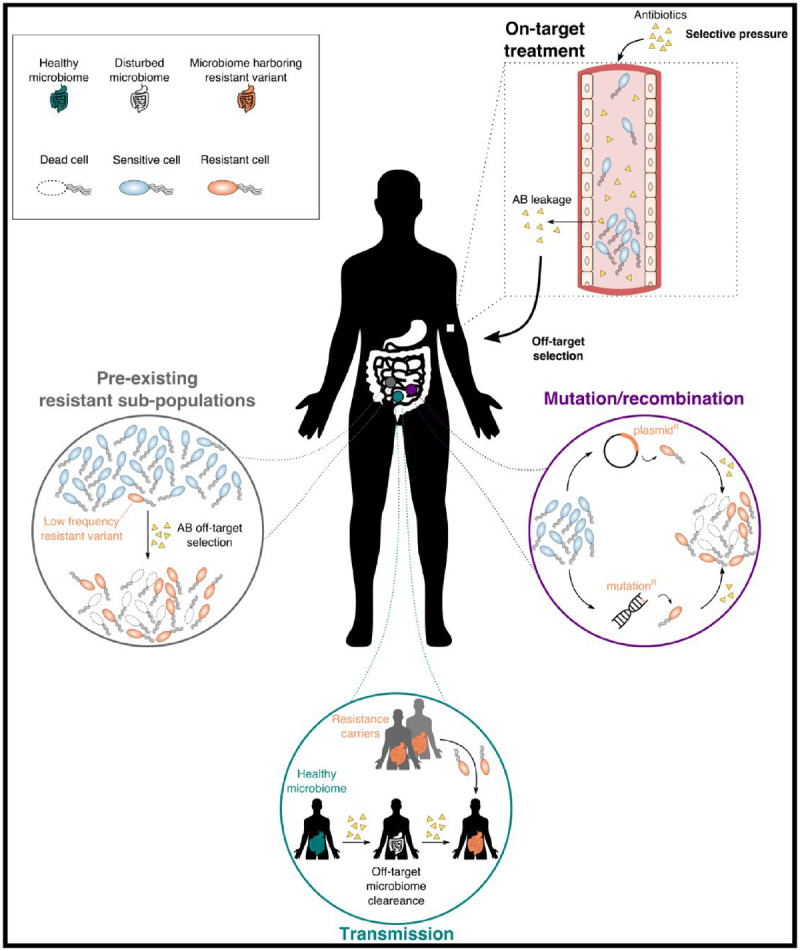
Evolutionary processes driving antibiotic resistance. The use of antibiotics can have undesired effects in off-target locations within a patient, with important consequences in terms of resistance evolution. We identify at least three potential population dynamics within these sites that can drive resistance evolution. The presence of antibiotics at these off-target sites selects for the emergence of resistance variants within a host via (i) *de novo* evolution of resistance through mutation or horizontal gene transfer (purple); (ii) pre-existing resistant sub-populations, whereby low-frequency resistant variants benefit from the clearance of competing sensitive cells or (iii) transmission of resistant variants from different hosts in a process known as colonization resistance

Antibiotic treatment is reactive: a patient presents with an infection and antibiotics that best treat the infection are prescribed. Antibiotic treatment is also causative: it modifies the microbial community in ways that impact the likelihood of future infection, the composition of those infections and their resistance phenotypes [1, 2]. Slowing the evolution of resistance within individual patients remains a challenge, in part, because physicians must balance the best treatment for the current infection, while minimizing their impacts at later stages. The rise of resistant organisms in a patient may follow any of three distinct pathways, each of which demands a different approach to antibiotic management (Fig. 1): (i) resistance may arise *de novo* as a result of mutation, recombination or horizontal gene transfer, (ii) antibiotic resistant organisms can be transmitted from one patient to another and (iii) an antibiotic resistant organism may already be present in the patient’s microbial community without evidently contributing to the current clinical infection.

**Figure 2.**
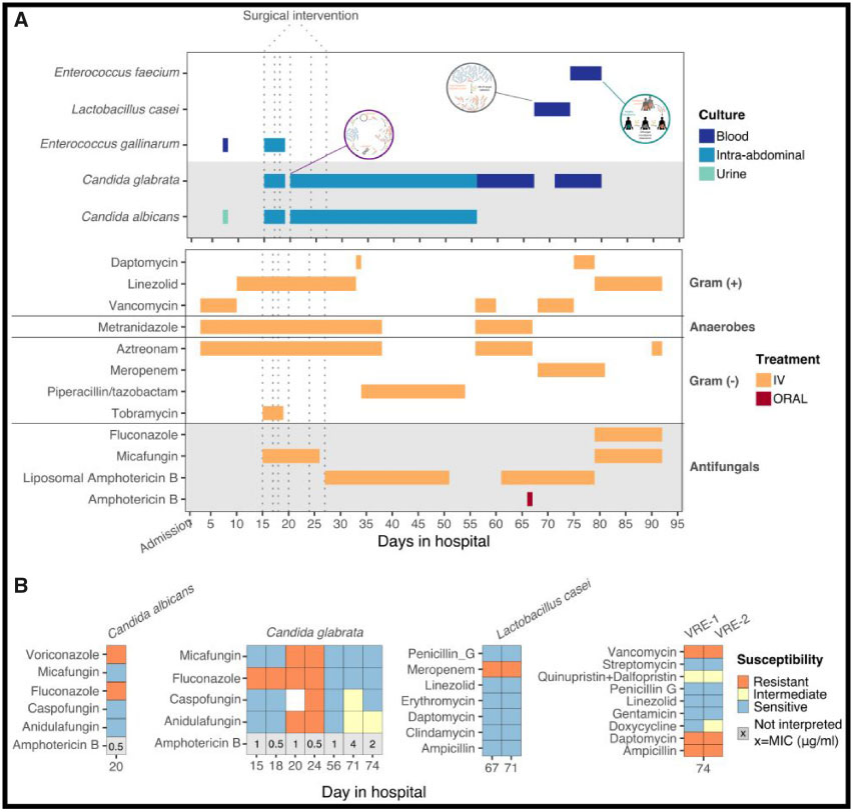
Patient’s course of infection (top panel) and antimicrobial treatment (lower panel) is shown in (A). Grey shaded areas highlight fungal infections and antifungal treatments. Vertical dotted lines correspond to days of surgical intervention. Antimicrobial treatment was given either orally (red) or intravenously (IV, in orange). Infections were identified from urine (light green), intra-abdominal fluid (blue) or blood (dark blue) cultures. Bubble stamps in the top panel emphasize infections where evidence suggests that antimicrobial resistance emerged through distinct evolutionary process. The ‘stamps’ are as depicted in Fig. 1. (B) Resistance profiles of the various infectious agents over time as determined by the Clinical and Laboratory Standards Institute (CLSI) breakpoints with MIC measured with Sensititre: Resistant (red), intermediate (yellow), sensitive (light blue) and non-interpretable MICs (grey; embedded x indicates the measured MIC in µg/ml)

Our goal is to describe the difficult task facing clinicians who must assess and respond to resistance evolution at the bedside. We present the case of a patient who survived an initial catastrophic illness, but then died following repeated infections by multi-drug resistant organisms. All three pathways leading to resistance emergence, which reflect distinct evolutionary and ecological dynamics, were implicated in this patient and lead to increasingly challenging infections and treatment dilemmas. The way forward must balance the impact of antibiotic therapy on each pathway in a manner that improves the patient’s health. Finally, we argue that medical providers at the bedside are best situated to perform the evolutionary risk assessment to identify optimal antibiotic choice, due to the complex and dynamic clinical situation and availability of real-time epidemiological data. However, they are often poorly equipped to make such an assessment due to lack of training in evolutionary principles, missing data and in some cases, incomplete theory. To convey these points, we first present the case in some detail (highlights are summarized in Fig. 2), and then present the evolutionary arguments in the discussion.

### Case presentation, management and outcome

A woman in her mid‐50s with no relevant past medical history presented to the emergency department with a 1-week history of upper abdominal pain that radiated to her back. Physical exam showed a distended abdomen, quiet bowel sounds and tenderness in the epigastric region and the right upper quadrant. Computed tomography (CT) imaging of the abdomen showed acute pancreatitis and she was admitted to the hospital. Over the next 24 h, despite adequate fluid resuscitation, her clinical status worsened with low blood pressures causing hypo-perfusion and multi-organ failure. She was transferred to the intensive care unit (ICU) on day 2 of hospitalization where she was intubated and started on mechanical ventilation. Intravenous vancomycin, aztreonam and metronidazole were started for empiric treatment of presumed bacterial infection leading to septic shock. Beta-lactam antibiotics were avoided because of a history of anaphylaxis to penicillin.

Timing of antibiotics and resistance profiles of bacteria and fungi are summarized in Fig. 2. On hospital day 7 the patient’s temperature rose to 38.4°C. Blood cultures grew *Enterococcus gallinarum*, which is intrinsically resistant to vancomycin. Vancomycin was discontinued, intravenous linezolid was started; aztreonam and metronidazole were continued. Blood cultures taken the following day were negative. A urine culture obtained with a Foley catheter in place grew *Candida albicans*. Her clinical status initially improved. She was extubated and moved to the general care ward.

On day 15, she became febrile, tachycardic, hypoxic, hypotensive, with exquisite abdominal pain. She was taken to the operating room for an exploratory laparotomy, which identified extensive necrosis of her terminal ileum, cecum, transverse colon and descending colon with perforation of the ileum and cecum, as well as gross spillage of gut contents into the abdominal cavity. Subtotal colectomy was performed with removal of a portion of the left and right colon and the ileum. Blood and peritoneal fluid cultures from day 15 both grew *Candida glabrata*, which was fluconazole resistant but susceptible to echinocandins (micafungin MIC (minimal inhibitory concentrations) 0.0015 µg/ml). The abdominal fluid also grew *C.albicans* and *E.gallinarum*. Intravenous micafungin was started; linezolid, aztreonam and metronidazole were continued. Intravascular catheters present at the time of fungemia were removed. Her blood cultures cleared on day 19.

She returned to the operating room five more times on days 17, 18, 20, 24 and 27. On day 27, exploratory laparotomy continued to identify necrotic exudate near the pancreas and murky fluid in the upper abdomen. Drains placed in the pancreatic bed and the right paracolic gutter on days 18 and 24 were left in place, and her abdominal wound was closed with mesh.

Abdominal fluid culture collected on day 20 grew *C.glabrata* that was resistant to fluconazole and newly resistant to echinocandins (micafungin MIC 2 µg/ml; fluconazole MIC 256 µg/ml) due to a point deletion in the *FKS2* gene hot spot 1, F659 (data not shown). *Candida albicans* resistant to fluconazole (MIC > 256 µg/ml) but sensitive to echinocandins also grew. Micafungin was discontinued and amphotericin B liposomal formulation was started. Abdominal fluid cultures from day 24 continued to grow *C.albicans* and similarly resistant *C.glabrata*.

After her final operation on day 27, an abdominal CT showed fluid collections abutting the pancreas and peritoneal enhancement, consistent with abdominal abscess and peritonitis. She developed renal failure requiring continuous renal replacement therapy on day 38. Her clinical status remained tenuous; the ICU team continued broad spectrum antimicrobials.

On hospital day 56 *C.glabrata* again grew from the blood. This isolate was susceptible to echinocandins, but given her history of resistant *Candida*, intravenous liposomal amphotericin B was restarted. Blood cultures cleared after 1 day. There was an attempt to de-colonize her gastrointestinal (GI) tract of resistant *Candida* by using oral amphotericin B (which has no systemic absorption), but this was stopped when gastrointestinal bleeding recurred, to avoid potentially exacerbating the bleeding.

Eleven days later the patient developed hypotension and increasing white blood cell count to 42.3 K/µL. Antibiotics were changed to vancomycin and meropenem. *Lactobacillus casei* resistant to both antibiotics grew in blood cultures. Vancomycin was replaced with linezolid, meropenem was continued, and *Lactobacillus* no longer grew from blood cultures after 7 days of therapy.

Concurrent with *L.casei*, the patient grew two isolates of *C.glabrata* from her blood on days 71 and 74. One was susceptible to high dose fluconazole (MIC 4 µg/ml), but had an increased MIC of 4 µg/ml to amphotericin B. The other isolate was susceptible to echinocandins but also had an elevated MIC of 2 µg/ml to amphotericin B. Liposomal amphotericin B was stopped due to concern that this MIC might convey clinical resistance (no clinical breakpoints exist); antifungal therapy was switched to intravenous micafungin and high-dose fluconazole.

On hospital day 74, blood cultures were positive for *Enterococcus faecium* resistant to vancomycin (VRE), ampicillin and daptomycin, but susceptible to linezolid and tigecycline. Linezolid treatment continued. Of note, on admission to the ICU a perirectal surveillance swab was negative for VRE; when rechecked at this time, it was positive. Central venous catheters were again exchanged. The patient’s blood cultures cleared of all organisms on hospital day 80. However, she had made no meaningful progress towards recovery, with continued respiratory, renal and liver failure. After discussion with family, comfort-focused measures were initiated on day 92. She died the following day.

## DISCUSSION

This case reveals the challenge of applying resistance management principles to clinical practice. While the pancreatitis that prompted the patient's initial presentation was not infectious in nature, her ensuing episodes of bloodstream infections developed increasing resistance and resulted in incremental clinical deterioration. Ideally, our understanding of resistance mechanisms would help us to prevent antibiotic resistant infections.

### Evolutionary principles arising during antimicrobial treatment

The patient’s initial clinical deterioration, necessitated broad spectrum therapy with vancomycin, aztreonam and metronidazole which eradicated drug-susceptible pathogens, including many in off-target spaces, such as the GI tract. When an antibiotic resistant organism is present at sub-clinical levels in the patient’s microbial community, the use of antibiotics to which it is resistant will facilitate its expansion through the elimination of competitors [3, 4]. Indeed, *L.casei*, *C.albicans* and *E.gallinarum* showed intrinsic resistance to initial broad spectrum therapy (Fig. 2B), despite being usually counted among the gut commensal bacteria and considered to have low pathogenicity [5]. In this case, it is not possible to prevent resistance from occurring; the focus should rather be to prevent those organisms from further proliferating to clinically significant levels within the patient and minimize the chance of transmission to others. Thus, the treatment must either be broad enough to cover these resistant organisms (and risk giving ‘too much’—a challenging proposition given the extensively resistant organisms that exist), or be as narrow as possible to preserve the antibiotic sensitive competitors (and risk giving ‘too little’—antibiotic sensitive bacteria may still harm the patient and contribute to resistance through *de novo* evolution) [4].

Alternatively, if resistance is not already present, it may arise *de novo* within a patient as the result of mutation, recombination or horizontal gene transfer. In diseases, such as HIV and tuberculosis, where *de novo* mutations are the greatest threat to successful treatment, history has shown it is best to follow Ehrlich’s advice: ‘hit hard and early’. This means dosing aggressively, potentially with multiple drugs, until the bacterial population is significantly diminished, thereby reducing the likelihood of any resistance mutation arising in the population [1, 6]. The resistance of *C.glabrata* to micafungin within the first 20 days of the patient’s hospitalization (Fig. 2A) provides the clearest example of *de novo* resistance emergence. The isolate was later confirmed to have a point mutation in *FKS2*. This mutation has previously been described to emerge under selection pressure, and almost exclusively found in patients with prior exposure to echinocandin antifungals [7, 8].

Finally, resistant organisms may arrive in the hospitalized patient through hospital-to-patient or patient-to-patient transmission. VRE is among the organisms the CDC has listed as a serious threat [9]. The vancomycin resistance was conferred by the vanA gene cluster, and generally acquired by transmission between patients, and known to be present in this hospital [10, 11]. In this case, VRE was not detected in an early surveillance screen performed upon the patient’s admission to the ICU, but was positive on a second swab some weeks later. When performed correctly, the perirectal VRE surveillance swab has >90% sensitivity [12]. While this cannot exclude the possibility that VRE was already at low levels when the patient was admitted, it is more likely that her second, positive swab signaled transmission from her environment. Antibiotic treatment against this pathogen can modify the risk of transmission by reducing colonization resistance of the recipient [13] through mechanisms that are yet incompletely understood [14]. It is often presumed that using antibiotics with narrow spectrum best preserves the ability of the existing microbial community to resist competitors [15], but even the use of narrow spectrum antibiotics may facilitate acquisition of an extensively drug resistant organism [16].

### Clinical decision-making needs to incorporate evolutionary risk assessments

From this case, we can see the challenges of antibiotic resistance management. The use of one antibiotic may lead to subsequent resistant infections through several mechanisms in different organisms. Unfortunately, even in retrospect is not possible to know with certainty which mechanisms were responsible. Moreover, optimal strategies to avoid resistance by one avenue may hasten resistance by another means. Finally, antibiotic options are often highly constrained by the patient’s clinical situation and the availability of culture data. There is often uncertainty about the microbial composition of the infection, and published data guiding the decision-making process in such cases have been incomplete [17].

Is it possible to do any better by identifying the most likely resistance pathway—*de novo*, transmission or emergence from a pre-existing source? In Box 1, we outline a concept map, based on evolutionary principles, to help medical providers think through possible routes of resistance emergence. We acknowledge that fundamental questions at every level of this conceptual framework remain unanswered at this time.

Box 1. **Conceptualization of an evolutionary risk assessment strategy.**

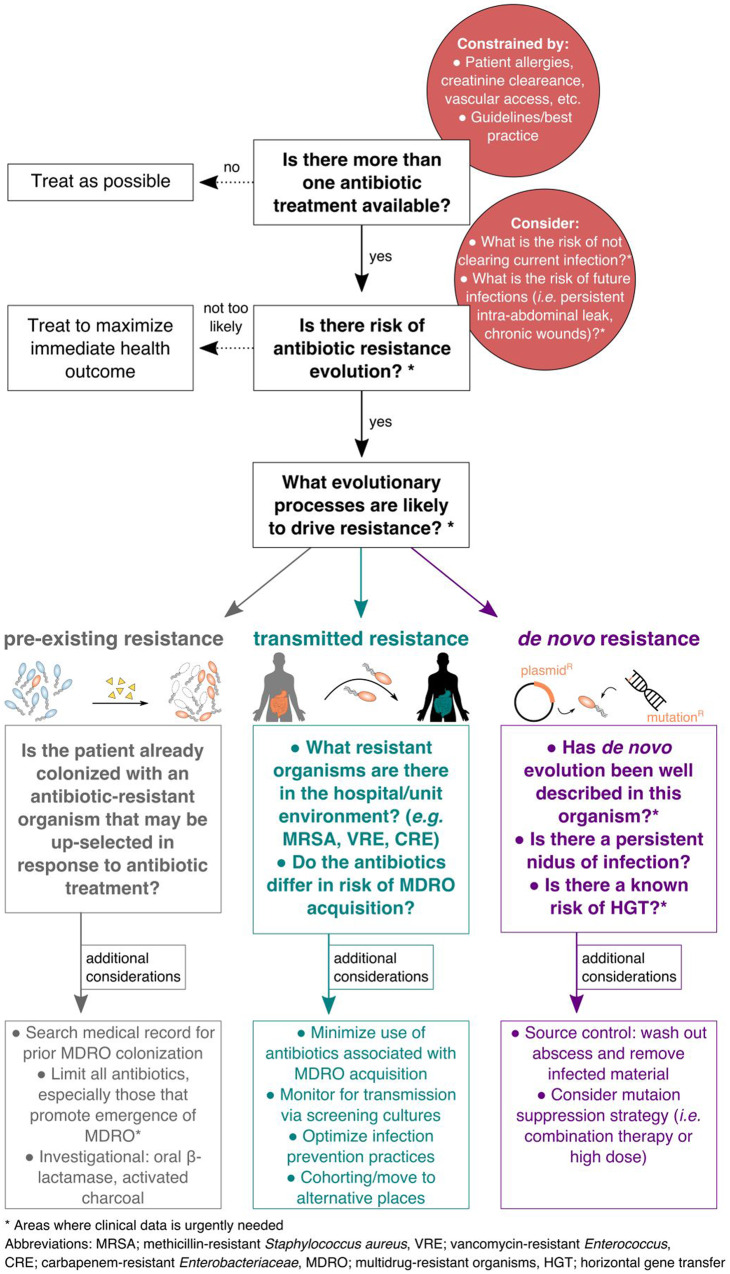
 Below we present a conceptual map that might aid clinical decision making in the light of evolutionary principles. At each level there are fundamental questions for which more data is still required, and specifically highlight those which are most critical. Note that the ideas put forward here are an initial conceptualization and do not represent official guidelines

## CONCLUSIONS

This case clearly demonstrates the reactive and causative sides of antibiotic therapy. The patient’s clinical course highlights all three routes to resistance and the clinical challenge of balancing these. Her case is far from unique in the complexity of infection, the number and duration of antibiotics or its ultimate conclusion. Ideally, clinicians would be able to estimate the risk of infection with a drug resistant organism for any patient before starting a course of antibiotics. This would allow them to make an informed decision that balances the immediate benefits of antibiotics with the long-term risk of resistance in the patient and onward transmission to the broader population. Factors influencing this assessment (Box 1) are rapidly changing, including the patient’s clinical status, culture data and physical location. We believe personalized risk assessment from the clinical team at the bedside will aid in antimicrobial decision making, but more data is desperately needed to improve those decisions.
